# Nitrate measurement and age-specific carcinogenic and non-carcinogenic risk assessment in commonly consumed vegetables from Khuzestan Province, Iran

**DOI:** 10.1038/s41598-026-40722-0

**Published:** 2026-02-25

**Authors:** Niloufar Pakravan, Seyyed Mohammad Ali Noori, Maryam Salehcheh, Mohammad Darvishnejad, Maryam Seyedtabib, Elaheh Mohammadi, Mohammad Javad Khodayar

**Affiliations:** 1 Toxicology Research Center, Medical Basic Sciences Research Institute, Ahvaz Jundishapur University of Medical Sciences, Ahvaz, Iran; 2 Department of Toxicology, School of Pharmacy, Ahvaz Jundishapur University of Medical Sciences, Ahvaz, Iran; 3 Department of Nutrition, School of Allied Medical Sciences, Ahvaz Jundishapur University of Medical Sciences, Ahvaz, Iran; 4 Nutrition Health Research Center, Food and Drug Control Laboratory, Hamadan University of Medical Science, Hamadan, Iran; 5 Infectious Ophthalmologic Research Center, Department of Biostatistics and Epidemiology, School of Health, Ahvaz Jundishapur University of Medical Sciences, Ahvaz, Iran

**Keywords:** Nitrate, Vegetables, Spectrophotometry, Risk assessment, Carcinogenic, Diseases, Environmental sciences, Risk factors

## Abstract

This study investigated nitrate concentrations and associated health risks in four commonly consumed vegetables in Iran. A total of 282 samples of cucumber, tomato, onion, and potato were randomly collected from markets across Khuzestan Province, and analyzed. Fresh vegetables were washed, homogenized, extracted, and analyzed using an optimized zinc-reduction Griess reaction-based spectrophotometric method (λ_max_ = 537 nm) validated against high-performance liquid chromatography (HPLC). The highest levels of nitrate were observed in potato (87.107 ± 32.270 mg/kg) and cucumber (80.366 ± 30.099 mg/kg). Non-carcinogenic risk assessment showed target hazard quotients (THQ) and hazard index (HI) well below 1.0 for both adults and children. Lifetime carcinogenic risk (CR) exceeded the conservative 1 × 10^−^⁶ threshold only for cucumber in adults and for cucumber, potato, and tomato in children. The total carcinogenic risk (TCR) was unacceptable. These findings suggest that, despite acceptable non-carcinogenic risk, long-term consumption of these vegetables may pose an unacceptable carcinogenic risk, particularly to children. Regular monitoring of nitrate levels in vegetables and other dietary sources (e.g., water and processed meat) is therefore recommended. However, some reducing agents in vegetables and our body can convert nitrate to the more toxic metabolite nitrite. Therefore, factors such as dietary composition and individual physiological responses to nitrate warrant further investigation to refine risk assessments.

## Introduction

Recent studies have raised significant public health concerns regarding pollutants in vegetables, including nitrates, heavy metals, and pesticide residues^1–2^. Dietary nitrate is largely converted to nitrite, which can form carcinogenic nitrosamines or cause methemoglobinemia in infants^3–7^. The European Food Safety Authority (EFSA) has established acceptable daily intakes (ADIs) of 3.7 mg/kg body weight for nitrate and 0.07 mg/kg body weight for nitrite^8–9^. Nitrate concentrations in vegetables and other plant-based foods vary widely depending on factors such as environmental conditions, plant species, nitrogen source, and storage duration^10^.

Despite the well-established role of vegetables as a major source of dietary nitrate, comprehensive year-round data for commonly consumed non-leafy vegetables remain limited. In Iran, non-leafy vegetables such as tomato, cucumber, potato, and onion constitute a significant part of the annual diet of the people. Khuzestan Province, located in southwestern Iran, is one of the main producers of these crops in the country. However, nitrogen fertilization and high evaporation rates in its hot climate facilitate the accumulation of nitrate in the soil. Despite numerous studies on vegetable nitrate in Iran^11–15^, comprehensive annual data for these four major non-leafy vegetables in Khuzestan are still scarce. Moreover, Khuzestan Province is the central hub of the oil and gas industry of the country, that its atmospheric sediments and industrial wastewater can be considered as secondary sources of nitrate in agricultural soils^16–17^. Previous studies in this province have been limited to single-season sampling, small sample sizes, or only the city of Ahvaz, and none has simultaneously assessed carcinogenic and non-carcinogenic risks to local consumers^18–19^.

Several analytical methods have been developed for nitrate detection^20–21^, then there is growing interest in simpler, faster, and more cost-effective approaches. The colorimetric Griess assay, first described in 1879, remains the most widely used method due to its simplicity and accessibility^22^. Although the Griess colorimetric method is widely used, conventional nitrate reduction methods rely on toxic cadmium or other hazardous reducing agents^23^. Recently, the zinc powder suggested as a safer alternative^24–25^.

Therefore, in this study, we developed and optimized a Griess-based colorimetric method using zinc powder reduction for nitrate determination in vegetables (tomato, cucumber, potato, and onion). The optimized method was validated against HPLC analysis. All samples were collected from local markets in Khuzestan Province. Additionally, both carcinogenic and non-carcinogenic health risks associated with dietary nitrate exposure were assessed statistically in children and adults population.

## Materials and methods

### Chemicals

Sulphanilamide, N-(1-naphthyl)-ethylenediamine dihydrochloride (NED), sodium nitrate, sodium nitrite, hydrochloric acid (HCl) (1.19 g/mL, 37%), zinc powder (Merck, Germany), acetonitrile (HPLC grade) and octylamine (99%, HPLC grade) were obtained from Daejung Co. Ltd. (South Korea) and Sigma Aldrich (St. Louis, MO, USA), respectively.

### Reagent solutions

Nitrate (NO₃^−^) and nitrite (NO₂^−^) stock solutions were prepared by dissolving analytical-grade sodium nitrate (NaNO₃) and sodium nitrite (NaNO₂) in deionized water (DI) to a concentration of 1000 mg/L (expressed as nitrate and nitrite ions). Standard solutions with lower concentrations (50, 25, 10, 5, 1, and 0.5 mg/L) were prepared by serial dilution of their respective stock solutions.

The Griess reagents were prepared as follows:

Sulfanilamide solution (2% w/v): 2 g of sulfanilamide was dissolved in 100 mL of 2.4 mol/L hydrochloric acid (HCl).

NED solution (0.2% w/v): 0.2 g of NED was dissolved in 100 mL of DI water.

Both reagent solutions were stored in amber bottles at 4 °C and remained stable for at least one month.

### Sampling

**Fig. 1:**
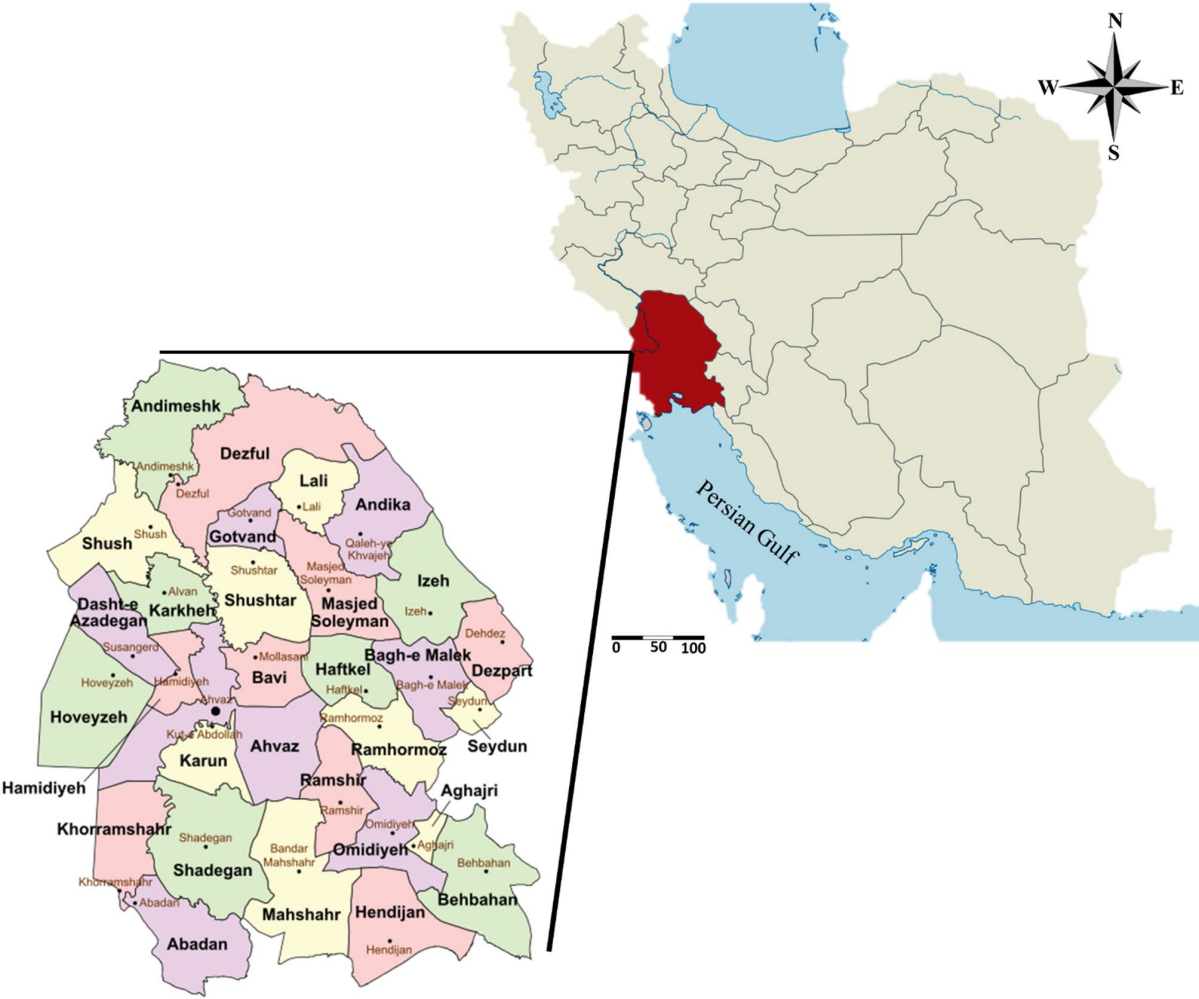
Map of the study area (Khuzestan Province, Southwestern Iran) showing geographic coordinates and the distribution of the sampling sites. Modified from a map obtained from Wikimedia Commons.

Vegetable samples (cucumber, tomato, onion, and potato) were collected from markets and retail stores across Khuzestan Province, Iran, between September 2022 and September 2023 (geographical distribution is shown in Fig. 1). Sampling was carried out over a 12-month period to capture seasonal variations and was conducted in collaboration with the Khuzestan Provincial Food and Drug Organization. A total of 282 samples were collected, comprising 75 cucumbers, 64 tomatoes, 72 onions, and 71 potatoes.

Sampling locations were selected using a random sampling method, with locations in each county distributed among wholesale markets and retail markets to ensure that these categories represented the majority of the province’s population. At each site, 3–5 separate retail units (approximately 1–2 plants each) of the same vegetable type and variety were purchased. After collection, samples were placed in clean containers, transported to the laboratory, and stored at 4 °C. Strategic combination of units ensured that each composite sample accurately represented regional consumption, while sampling at multiple independent sites provided strong provincial coverage.

### Sample preparation

The vegetable samples were washed with tap water and then DI water before processing. The edible portions of the samples were collected, cut, and homogenized. Then, the samples were divided into two parts for spectrometric and HPLC analyses and immediately stored at − 20 °C before the analysis.

### Spectrophotometric determination of nitrate

Nitrate was determined spectrophotometrically using a modified Griess assay based on ISO 6635:1984, in which nitrate is first reduced to nitrite with zinc powder.

Briefly, 10 g of homogenized vegetable sample was transferred to a 100 mL Erlenmeyer flask, and 50 mL of hot DI water (80 °C) was added. The mixture was heated in a water bath at 80 °C for 10 min with occasional swirling. After cooling to approximately 40 °C, 0.2 g of zinc powder was added as the reducing agent.

The extract was quantitatively transferred to a 50 mL volumetric flask, made up to volume with DI water, and centrifuged at 4000 × g for 10 min at 4 °C. An aliquot of the clear supernatant (2 mL) was transferred to a test tube, followed by sequential addition of 0.5 mL of sulfanilamide solution (2%) and 0.5 mL of NED solution (0.2%). After gentle mixing, the mixture was incubated in the dark for 5 min to allow full color development. Absorbance of the resulting pink azo dye was measured at 537 nm by an ultraviolet visible spectrophotometer (Shimadzu 2401PC UV-VIS Spectrometer, Kyoto, Japan). The nitrate content was calculated with a calibration curve.

### Preparation of calibration curve

The calibration curve for nitrite was set at 0.5, 1, 5, 10, 25, and 50 mg/L of NO_2_^−^ and NO_3_^−^ solution. Nitrate was previously reduced to nitrite by zinc powder. Each of the above concentrations was transferred into a separate 50-mL volumetric flask, and 0.5 mL of sulfanilamide was added and mixed. Then, 0.5 mL of NED was added, and, after 5 min, the absorbance was measured at 537 nm.

### HPLC method

Nitrate was also quantified by reversed-phase ion-pair HPLC for method validation. For the nitrate content measurement by HPLC [Waters, FLD 2475, Detector 2487, Phenomenex Luna C18 HPLC column (5 m, 250 × 4.6 mm i.d.)], the homogenized sample was prepared as in the previous step. After centrifugation, a portion of the supernatant was passed through a 0.45 μm PTFE syringe filter and injected immediately (within 1 h of preparation) to avoid any degradation of nitrate. The condition of the mobile phase (water 80%, acetonitrile 20%, octylamin 1.6%, pH 4.5 adjusted by orthophosphoric acid, and flow rate 1 mL/min) was used in the experiment. The nitrate content was calculated using the nitrate calibration curve.

### Spectrophotometric method validation

To validate the method, the precision (RSD%), accuracy (recovery%), calibration curve, linearity of the calibration curve, limit of detection (LOD), limit of quantification (LOQ), and real sample analysis follow the guidelines set by the International Conference on Harmonization Guidelines^27^. The calibration curve was constructed using different nitrate standards in the range of 0.5–50 mg/kg.

For spike analysis, the homogenized sample was divided into two equal portions. One portion was processed using the method described earlier for nitrate measurement. The other portion was mixed with a nitrate standard solution (10 mg/L), processed using the same method, and analyzed to determine the recovery rate. The recovery percentage, LOD, and LOQ were calculated using Eqs. 1, 2, and 3, respectively.

1 $$\:\mathrm{R}\:\left(\mathrm{\%}\right)=Cmeas/Cspk\times\:100$$

R (%): recovery percentage or accuracy; C_meas_.: concentration of measured nitrate; C_spk_: concentration of spiked nitrate.

2 $$\:\mathrm{L}\mathrm{O}\mathrm{D}=3.3\times\:SD/S$$

3 $$\:\mathrm{L}\mathrm{O}\mathrm{Q}=10\times\:SD/S$$

LOD: limit of detection; LOQ: limit of quantification; SD: standard deviation of the blank sample; S: slope of the standard curve.

Precision was assessed by performing six consecutive evaluations of nitrate within three consecutive days, both intra-day and inter-day data were analyzed (Eq. 4) ^28^.

4 $${\text{RSD~\% }}=SD/\bar {X} \times 100$$

RSD %: relative standard deviation percentage or precision; SD: standard deviation of samples;$\bar {X}$ : mean of samples.

### Non-carcinogenic health risk assessment

#### Estimated daily intake

In this study, the classification of nitrates as non-carcinogenic chemicals (IRIS 1991) was employed to assess the potential non-carcinogenic health risks associated with vegetable consumption for both adults and children. The assessment for adults was conducted using Eq. 5 ^29^, but in children based on their daily consumption, which is half that of adults, and the average body weight (children: 20 kg, adults: 70 kg)^30^, children’s EDI was calculated by multiplying the adult’s EDI by 1.75 (obtained from $\:70/20\:\times\:\:1/2)$.

5 $$\:\mathrm{E}\mathrm{D}\mathrm{I}\:\mathrm{a}\mathrm{d}\mathrm{u}\mathrm{l}\mathrm{t}\mathrm{s}=\frac{MC\:\times\:\:DC}{BW}\:\mathrm{E}\mathrm{D}\mathrm{I}\:\mathrm{c}\mathrm{h}\mathrm{i}\mathrm{l}\mathrm{d}\mathrm{r}\mathrm{e}\mathrm{n}=\mathrm{E}\mathrm{D}\mathrm{I}\:\mathrm{a}\mathrm{d}\mathrm{u}\mathrm{l}\mathrm{t}\mathrm{s}\times\:1.75$$

EDI: estimated daily intake (mg/kg/day); MC: mean concentration of nitrate (mg/kg wet weight); DC: daily consumption (cucumber: 0.109, tomato: 0.109, onion: 0.039, and potato: 0.068 kg/day for Iranian adult)^31^; BW: mean body weight (adult: 70 kg)^32^.

#### Hazard quotient

Non-carcinogenic risk is evaluated using the Target Hazard Quotient (THQ) (Eq. 6)^33^:

##### Equation 6

THQ =$$\:EDI/RfD$$.

THQ: target hazard quotient; EDI: estimated daily intake (mg/kg/day); reference dose (RfD): according to the risk information system recommended by the United States Environmental Protection Agency (USEPA), the reference dose for nitrate is 1.6 mg/kg/day^34, 35^.

If the THQ is less than one, it indicates that the chronic health risk is within an acceptable range; however, a THQ greater than one indicates non-acceptable level of the non-carcinogenesis risk^36^. To estimate the non-carcinogenesis risk of nitrates in different types of vegetables, the hazard index (HI) was used. The HI is calculated based on Eq. 7, and n means types of vegetables^37^:

7 $$\:\mathrm{H}\mathrm{I}={\sum\:}_{n=1}^{i}THQ\:n;i=1,\:\:2,\:\dots\:,\:n$$

### Carcinogenic health risk assessment

The acceptable limit of carcinogenic risk (CR) is 1 × 10^− 6^ and a CR greater than 1 × 10^− 6^ is a non-acceptable limit^38^. Cancer slope factors (CSF)^39^ are utilized to assess the risk of cancer associated with exposure to a carcinogenic or potentially carcinogenic substance. The following equation was used to calculate the risk of cancer (Eq. 8)^40^:

8 $${\mathrm{CR}}\,=\,{\mathrm{EDI}} \times {\mathrm{CSF}}$$

CR: carcinogenic risk; EDI: estimated daily intake (mg/kg/day); CSF: cancer slope factor, which is 10^− 5^ mg/kg/day for nitrate^41^.

Therefore, Eq. 9 carried out the calculation of the total carcinogenic risk (TCR), which could have a carcinogenic effect based on exposure dose in different types of vegetables, and n means types of vegetables^42^:

9 $$\:\mathrm{T}\mathrm{C}\mathrm{R}={\sum\:}_{n=1}^{i}CR\:n;i=1,\:\:2,\:\dots\:,\:n$$

### Statistical analysis

All data were performed in triplicate in independent experiments. To examine the normality of the data, a Kolmogorov-Smirnov test was used. Quantitative data are expressed as means± standard deviation (SD). For the abnormal distribution of nitrate in the samples, nonparametric statistical tests were used for the analyses, using the Wilcoxon test. Meanwhile, the normally distributed group analysis of variance (ANOVA) was determined by using GraphPad Prism 9.0 software. P values less than 0.05 were statistically significant.

## Results and discussion

### Spectrophotometric method validation

The LOD and LOQ of nitrate in the vegetables in this study were obtained as 4.590 mg/kg and 13.910 mg/kg, respectively (Table 1). These values confirm that the optimized zinc-reduction Griess method is sufficiently sensitive for routine monitoring of nitrate in vegetables at the range of regulatory or health-based guidance levels^43^.

**Table 1:** Linear equation and regression coefficient of calibration curves and detection limit (LOD) and quantitative limit (LOQ) nitrate of the spectrophotometric method.

	Equation	Regression coefficient	LOD (mg/kg)	LOQ (mg/kg)
**Nitrate**	y = 0.032x + 0.063	0.998	4.590	13.910

The precision and accuracy data for the optimized method are summarized in Table 2. Based on the results, the lowest RSD was 1.023% (inter-day) and 0.950% (intra-day) from the onion sample, and the highest RSD was 3.286% (inter-day) and 2.980% (intra-day) from the cucumber sample. The higher RSD observed in cucumber is likely due to its high water content (95–97%), which may cause minor matrix variations during hot water extraction. Nevertheless, it remains within the acceptable range for food analysis (< 5%) ^44–46^. The total RSD for all groups was less than 6%, which indicated the stability of the vegetable samples. In addition, the recovery (R%) results are shown in Table 2. The lower recovery analysis of spiked samples was observed in potatoes at 87.520% (inter-day) and 83.460% (intra-day). However, higher recovery rates were observed for tomato and onion, suggesting that the lower recovery observed for potato may be due to matrix effects. The results showed the accuracy and precision values for nitrate, with the lowest average recovery (87.520%) and largest RSD% (2.980%).

Consequently, the levels of nitrate in 282 vegetable samples, namely cucumber, potato, tomato, and onion, were assessed. The concentrations of nitrate were measured to be 80.366 ± 30.099, 58.051 ± 18.602, 53.830 ± 15.777, and 87.107 ± 32.270 mg/kg in cucumber, tomato, onion, and potato, respectively. In an earlier study conducted in Ahvaz (Khuzestan Province), Veissi et al.^47^ reported mean nitrate concentrations of 104.70 ± 1.81 in potato and only 7.37 ± 0.16 in tomato. However, the average amounts of nitrate reported by Mehri et al. in Hamadan Province, western Iran, who recorded mean values, were higher than the results obtained in this study, with onion exhibiting a mean of 82.25 ± 92.22 mg/kg, potato 129.30 ± 86.51 mg/kg, and tomato 110.93 ± 166.08 mg/kg^41^. The relatively low nitrate concentration observed in Khuzestan, compared to Hamedan Province, is likely due in part to the region’s hot and dry climate and intense solar radiation. These conditions accelerate plant growth, enhance nitrate uptake by increasing the activity of the nitrate reductase enzyme, and ultimately reduce nitrate accumulation in edible tissues^48–49^. Similarly, the findings of this study indicated that the mean concentration of nitrate observed in vegetables was comparatively lower than those documented in previous research^37,50^. As previously stated, nitrate accumulation in vegetables is affected by other factors, including the amount and quality of fertilizer, the timing of fertilization, soil type, and harvest time^51, 52^. In addition, the predominance of calcareous and alkaline soils in Khuzestan Province, along with high rates of evapotranspiration, reduces the mobility and leaching of nitrate, ultimately decreasing the amount of nitrate available for plant uptake, compared to acidic or neutral soils in the northern and western regions of Iran^53, 54^.

**Table 2:** The precision and recovery of vegetable samples in spectrophotometric analysis.

Vegetable				Inter-day		Intra-day	
	*N*	M	Spiked level (mg/kg)	RSD (%)	*R* (%)	RSD (%)	*R* (%)
Cucumber	3	6	10	3.286	92.600	2.980	88.570
Tomato	3	6	10	2.141	108.990	2.210	91.450
Onion	3	6	10	1.023	106.300	0.950	92.270
Potato	3	6	10	1.126	87.520	1.010	83.460

N: analysis days; M: the number of analyses per day; RSD (%): precision; R (%): recovery.

### The nitrate content of vegetables

The azo dye formed by the reaction of nitrite with the Griess reagent exhibited a maximum absorbance at a wavelength of 537 nm, which is consistent with previous reports for this colorimetric assay. Nitrate concentrations in the 282 vegetable samples are summarized in Table 3. Mean values ranged from 53.830 ± 15.777 mg/kg (onion) to 87.107 ± 32.270 mg/kg (potato), with intermediate levels in cucumber (80.366 ± 30.099 mg/kg) and tomato (58.051 ± 18.602 mg/kg). Considerable variation was observed within each vegetable type (CV 35–52%), reflecting differences in cultivation practices, soil conditions, and seasonal effects across Khuzestan Province.

All mean nitrate concentrations were well below the maximum limits established by the Iranian National Standard No. 16,596^43^, for these vegetables (Table 3). Compared with earlier studies conducted in the same region, the levels reported here are notably lower. For example, Veissi et al.^47^ reported mean concentrations of 104.7 ± 1.8 mg/kg in potato and only 7.4 ± 0.2 mg/kg tomato in Ahvaz, whereas Mehri et al.^41^ found considerably higher values (onion: 82.3 ± 92.2 mg/kg, potato: 129.3 ± 86.5 mg/kg, tomato: 110.9 ± 166.1 mg/kg). The lower nitrate contents observed in the present study are in closer agreement with more recent surveys from other Iranian provinces^37,50^. These findings indicate that industrial factors, including atmospheric deposition and wastewater, appear not to have significantly affected nitrate accumulation in the vegetables studied. As widely documented, nitrate accumulation in vegetables is strongly influenced by multiple factors, including fertilizer type and application rate, soil characteristics, irrigation practices, light intensity, temperature, planting density, and harvest timing^51, 52^.

**Table 3:** The spectrophotometric measured nitrate content in vegetables.

Samples	*N*	Mean (mg/kg)	SD	Minimum	Maximum	MAC
Cucumber	75	80.366 ± 30.099	30.099	20.001	142.383	300
Tomato	64	58.051 ± 18.602	19.682	16.214	90.918	150
Onion	72	53.830 ± 15.777	15.777	17.072	87.767	90
Potato	71	87.107 ± 32.270	31.860	26.465	158.980	250

SD: Standard deviation; Maximum Allowable Concentration (MAC) according to Iranian National Standardization Organization^43^.

### Validation of spectrophotometric method

**Fig. 2:**
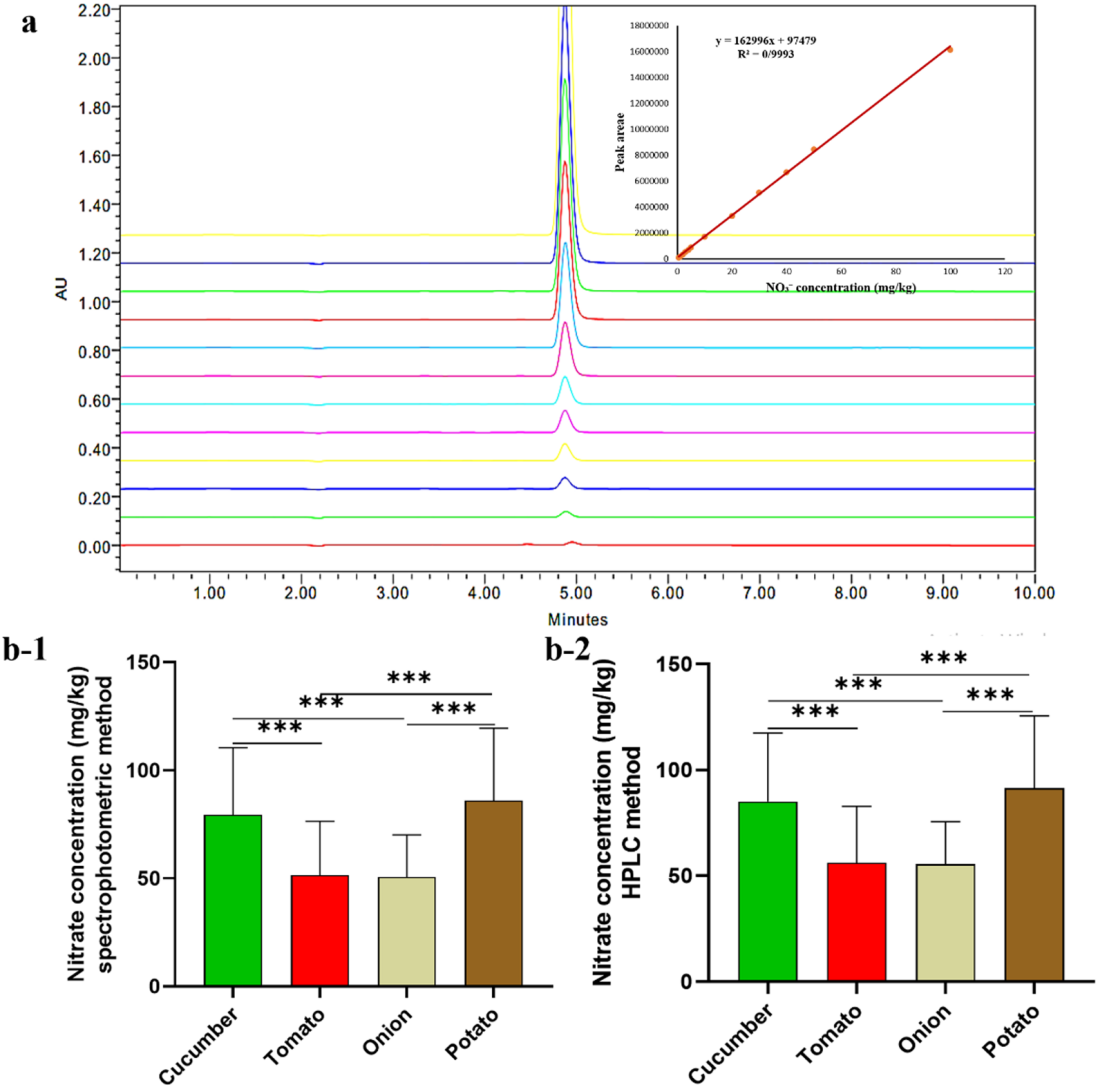
Comparison of the spectrophotometric and HPLC methods of nitrate analysis; a HPLC standard concentration chromatogram and HPLC calibration curve; b-1 The spectrophotometric and b-2 the HPLC methods of nitrate analysis in cucumber, tomato, onion, and potato. ***p < 0.001.

**Fig. 3:**
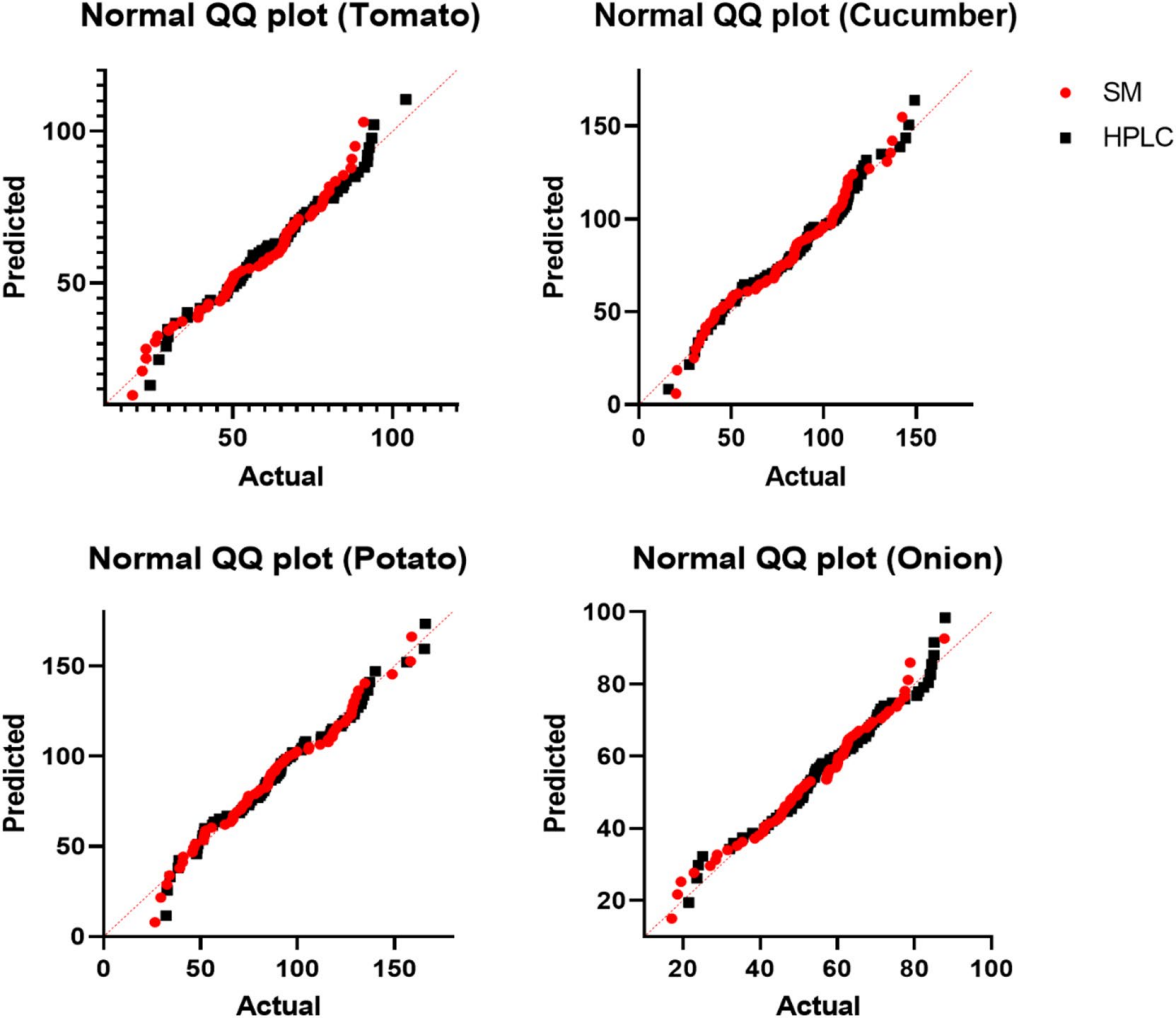
Comparison and validation of the spectrophotometric method (SM) against HPLC method of nitrate analysis, by the quantile-quantile (Q-Q) plot comparison in cucumber, tomato, onion, and potato.

For validation of the spectrophotometric method studied against the HPLC method, the nitrate content of all four vegetables (cucumber, tomato, onion and potato) was determined by both spectrophotometric and HPLC methods (Fig. 2). In addition, the average nitrate concentrations in spectrophotometry and HPLC analysis of cucumber, tomato, potato, and onion were 80.366 ± 30.099 and 86.065 ± 31.423, 58.051 ± 18.602 and 63.407 ± 19.441, 87.107 ± 32.270 and 92.538 ± 32.990, and 53.830 ± 15.777 and 58.882 ± 16.057 mg/kg, respectively. According to the obtained results, in all products, the spectrophotometric method showed a lower nitrate content than the HPLC method, with a significant difference between these mean values (*P* < 0.001). This difference is probably due to minor errors caused by impurity interference with the samples in the spectrophotometric method. The spectrophotometric method’s nitrate content was nearly 7% lower than the HPLC method. In this study, there is a linear regression relationship between these two methods, and the intraclass correlation of spectrophotometric and HPLC methods is shown in Fig. 3; Table 4. Therefore, this method demonstrates good reliability for high-throughput monitoring, and HPLC is recommended as a confirmatory analytical tool for high-risk samples.

**Table 4:** Intraclass correlation coefficient of spectrophotometric and HPLC methods.

Vegetable type	ICC*
Cucumber	0.994
Tomato	0.985
Onion	0.980
Potato	0.996

*Intraclass correlation coefficient.

### Non-carcinogenic health risk assessment findings

Estimated daily intake (EDI) of nitrate and the corresponding target hazard quotients (THQ) for adults and children are summarized in Table 5. In this study, nitrate EDI values for adults and children followed the order: cucumber> potato> tomato> onion. The cucumber exhibited the greatest EDI values of 0.125 and 0.219 mg/kg body weight per day in adults and children, respectively. Whereas, the onion sample displayed the lowest EDI values of 0.030 and 0.052 mg/kg body weight per day in adults and children, respectively. According to World Health Organization (WHO) guidelines, the EDI threshold for all vegetable samples was less than the authorized level for both adults and children, indicating that none of the entities tested exceeded the specified threshold. Consequently, in the study by Uddin et al.^50^ the tomato EDI exhibited a lower value (0.17 and 0.37 mg/kg body weight per day in adults and children), whereas the potato EDI had a greater value (1.19 and 2.16 mg/kg body weight per day in adults and children), respectively.

Cucumber has the highest nitrate content among the tested vegetables for adults and children, followed by potatoes and tomatoes. All individual THQ values were less than one (maximum 0.137 for cucumber in children), indicating negligible non-carcinogenic risk from consumption of any single vegetable. The study’s findings showed that the nitrate THQ was well below the index, indicating that these vegetarians are not a cause for concern. In adults and children, cucumber had the maximum nitrate THQ, while onion had the lowest amount. However, for both adults and children, the order of nitrate THQ was as follows: cucumber, potato, tomato, and onion. Therefore, calculating THQ for one source alone does not give a complete picture of the overall risk to the body. To make a more accurate assessment, the total nitrate intake from all sources must be calculated, which is a difficult task. Since in this study, The calculation was performed to determine the cumulative risk of four commonly consumed vegetables in the Iranian population, using the HI^55,56^. The HI, representing cumulative non-carcinogenic risk from simultaneous consumption of all four vegetables, was 0.206 for adults and 0.362 for children, well below the safety threshold of one. Therefore, the consumption of these four vegetables does not pose a significant health risk or non-carcinogenic danger to the local population. However, daily intake directly correlates with nitrate toxicity, and factors such as consumption rate, intrinsic risk, and body weight determine the health risk of nitrates.

**Table 5:** Estimated daily intake (EDI) and health target hazard quotient (THQ) for vegetable samples according to current daily intake for adults and children.

Samples	According to current daily consumption			
	Nitrate EDI mg/kg body weight/day		THQ	
	Adult	Children	Adult	Children
Cucumber	0.125	0.219	0.078	0.137
Tomato	0.090	0.158	0.056	0.099
Onion	0.030	0.052	0.019	0.033
Potato	0.085	0.148	0.053	0.093
HI	–	–	0.206	0.362

EDI: estimated daily intake; THQ: target hazard quotient; HI: hazard index.

### Carcinogenic risk assessment data

The lifetime excess CR values due to dietary nitrate exposure are reported in Table 6. The acceptable value for CR is considered to be 1 × 10^−^⁶, and if the CR value or TCR exceeds 1 × 10^−^⁶, it is regarded as being in the unacceptable range^40, 57^. The results showed that the CR values for cucumber in adults and children were 1.250 × 10^−^⁶ and 2.190 × 10^−^⁶, respectively, indicating an undesirable and unacceptable level of carcinogenic risk. In addition, the CR values for tomatoes and potatoes in children were 1.580 × 10^−^⁶ and 1.480 × 10^−^⁶, respectively, indicating an unacceptable carcinogenic risk for this age group. Accordingly, the TCR results also demonstrated a significant association between the consumption of these vegetables and potential carcinogenic risk (TCR > 1 × 10^−^⁶). Overall, the potential carcinogenic risk from the combined consumption of these vegetables is not within acceptable limits, and their cumulative effects should not be ignored.

**Fig. 4:**
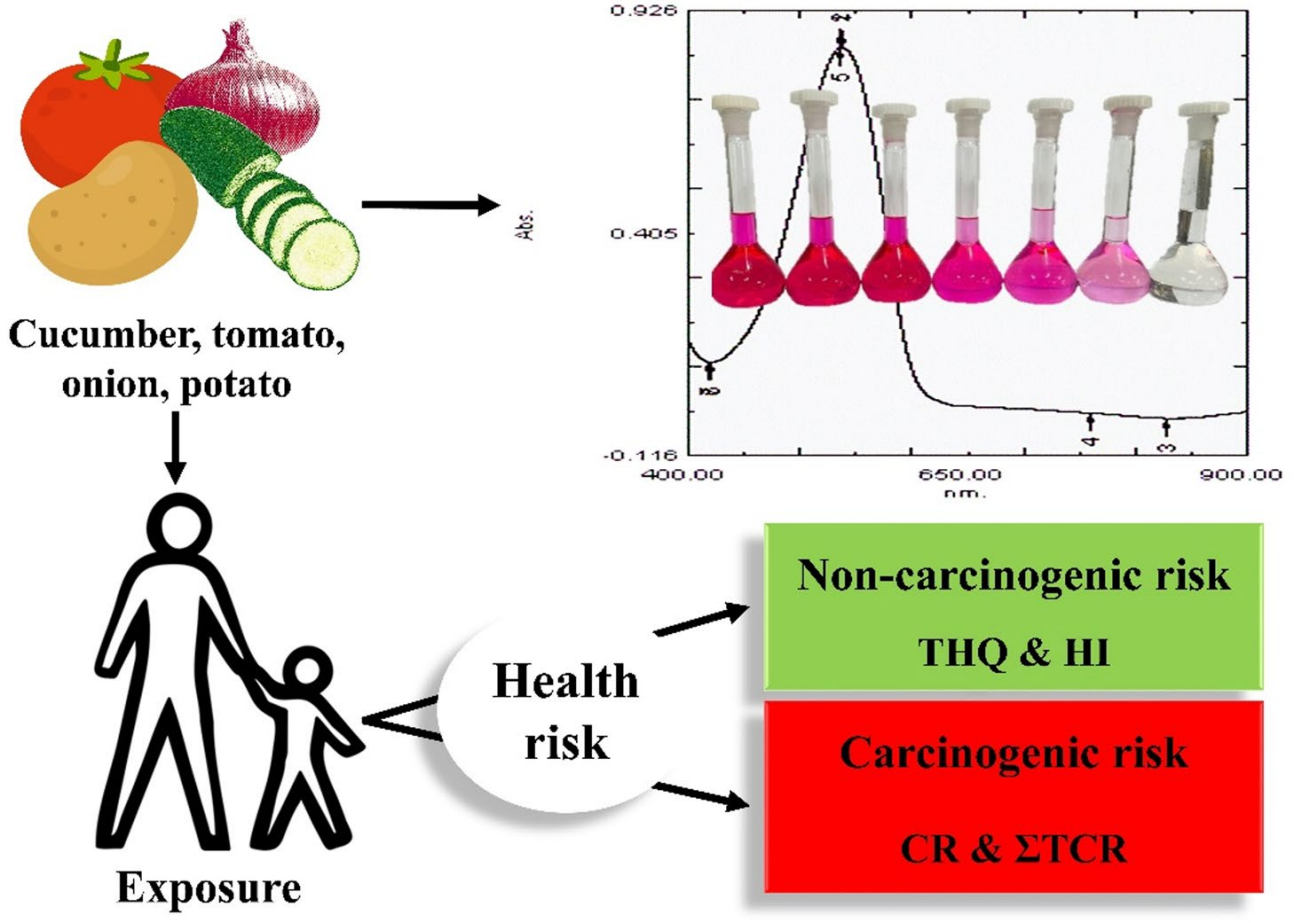
The graphical abstract illustrates the process of nitrate measurement and risk assessment for human health. The nitrate content in cucumber, tomato, onion, and potato harvested from Khuzestan Province, Iran, was determined using an optimized zinc reduction-based Griess spectrophotometric method. The results showed that the non-carcinogenic risks (THQ and HI) were in a negligible range and less than 1.0, while the carcinogenic risk was estimated to be slightly above the conservative threshold of 10^−6^.

Based on the findings of this study, the CR and TCR values related to nitrate in vegetable samples, in both adults and children, were assessed as unacceptable. Therefore, continuous monitoring is recommended, especially for the pediatric population. These results indicate that the nitrate content in these vegetables is not desirable for human health, and their long-term consumption may pose serious health risks. Consequently, this study suggests that the potential carcinogenic risk associated with nitrate in these vegetables in Khuzestan Province, Iran, may be of concern. On the other hand, studies have shown that nitrate can induce antioxidant and mitochondrial responses and modulate inflammatory reactions and immune cell function 58, 59. In addition, some compounds such as ascorbic acid, ferulic acid, caffeic acid, α-tocopherol, and certain amino acids, including glycine, lysine, and histidine, are able to inhibit the formation of nitrosamines from nitrate and nitrite 60. Moreover, these findings indicate that factors such as dietary pattern, lifestyle, and vegetable intake can also influence the cancer risk associated with the consumption of these foods. Figure 4 also graphically presents the results of the present study.

**Table 6:** Nitrate carcinogenic risk levels for adults and children, based on the carcinogenicity slope factor.

Vegetable type	CR		CSF (mg/kg/day)
	Child	Adult	
Cucumber	2.190 × 10^− 6^*	1.250 × 10^− 6^*	10^− 5^
Tomato	1.580 × 10^− 6^*	0.900 × 10^− 6^	10^− 5^
Onion	0.520 × 10^− 6^	0.300 × 10^− 6^	10^− 5^
Potato	1.480 × 10^− 6^*	0.850 × 10^− 6^	10^− 5^
TCR	5.770 × 10^− 6^*	3.300 × 10^− 6^*	

CR: carcinogenic risk; TCR: total carcinogenic risk; CSF: carcinogenicity slope factor.

* CR and TCR greater than 1 × 10^− 6^, indicated the carcinogenic risk is higher than the acceptable limit.

Although this study provides a comprehensive annual assessment of nitrate levels in the most important non-leafy vegetables across Khuzestan Province, several limitations should be noted. The sampling strategy was reflective of consumer exposure but did not account for possible variations at the farm level. Only raw vegetables available in the market were analyzed, although post-harvest processing can reduce nitrate levels, therefore, the actual dietary intake is likely to be lower than the estimated amount. Health risk estimates were based solely on these four vegetables and did not include other important sources such as drinking water and processed meats, which could lead to an underestimation of total exposure and an overestimation of the relative contribution of vegetables to carcinogenic risk. Carcinogenic risk calculations were also based on conservative carcinogenicity slope coefficients, although endogenous nitrosation is reduced by dietary inhibitors that are abundant in the Iranian diet; this suggests that the true risk is likely lower. Finally, the Griess method based on zinc reduction in samples with high water content showed a slight negative bias, suggesting that confirmatory analysis by HPLC is recommended for regulatory purposes. These limitations are common features of market basket studies and emphasize the need for future research, including farm-to-table nitrate reduction strategies and integrated exposure modeling.

## Conclusion

Based on the obtained values in this study, it was concluded that the average nitrate concentration in all vegetable samples was below the established permissible limit. The non-cancer risks associated with nitrate were less than one. However, the carcinogenic risk was at an unacceptable level that could lead to adverse health effects. Considering that vegetables are the main source of human exposure to nitrate, it is also particularly important to investigate the ecological and environmental distribution of nitrate from alternative sources such as water and animal products.

## Data Availability

The data that supports the findings of this study are available from the corresponding author upon reasonable request.
